# Testing the ability of unmanned aerial systems and machine learning to map weeds at subfield scales: a test with the weed Alopecurus myosuroides (Huds)

**DOI:** 10.1002/ps.5444

**Published:** 2019-05-21

**Authors:** James PT Lambert, Dylan Z Childs, Rob P Freckleton

**Affiliations:** ^1^ Department of Animal & Plant Science University of Sheffield Sheffield U.K.

**Keywords:** unmanned aerial systems, weed mapping, convolutional neural networks, black‐grass, management

## Abstract

**BACKGROUND:**

It is important to map agricultural weed populations to improve management and maintain future food security. Advances in data collection and statistical methodology have created new opportunities to aid in the mapping of weed populations. We set out to apply these new methodologies (unmanned aerial systems; UAS) and statistical techniques (convolutional neural networks; CNN) to the mapping of black‐grass, a highly impactful weed in wheat fields in the UK. We tested this by undertaking extensive UAS and field‐based mapping over the course of 2 years, in total collecting multispectral image data from 102 fields, with 76 providing informative data. We used these data to construct a vegetation index (VI), which we used to train a custom CNN model from scratch. We undertook a suite of data engineering techniques, such as balancing and cleaning to optimize performance of our metrics. We also investigate the transferability of the models from one field to another.

**RESULTS:**

The results show that our data collection methodology and implementation of CNN outperform pervious approaches in the literature. We show that data engineering to account for ‘artefacts’ in the image data increases our metrics significantly. We are not able to identify any traits that are shared between fields that result in high scores from our novel leave one field our cross validation (LOFO‐CV) tests.

**CONCLUSION:**

We conclude that this evaluation procedure is a better estimation of real‐world predictive value when compared with past studies. We conclude that by engineering the image data set into discrete classes of data quality we increase the prediction accuracy from the baseline model by 5% to an area under the curve (AUC) of 0.825. We find that the temporal effects studied here have no effect on our ability to model weed densities. © 2019 The Authors. Pest Management Science published by John Wiley & Sons Ltd on behalf of Society of Chemical Industry.

## 1 INTRODUCTION

The core objective of plant population ecology is to understand changes in numbers of individuals/organisms across time and space.^1^ Achieving this depends on methods that permit plants to be mapped and monitored at informative scales.^2^, ^3^, ^4^ Surveys of plant populations have been undertaken using a variety of different methods such as transect sampling, quadrat sampling and with unmanned aerial systems (UAS).^5^, ^6^, ^7^ Each of these methods has an inherent trade‐off between the area that can be surveyed and the intensity at which the subjects in that area can be studied.^8^ Transect and quadrat sampling can be used for either small area, high‐intensity studies or large area, low‐intensity studies, but typically not both.^9^

UAS present a unique opportunity for ecological monitoring because, potentially, they can yield data across both large spatial areas and at high survey intensity. This bridges the gap between local scales at which interactions matter, and larger landscape scales at which environmental variation is important.^10^ UAS have been applied in a range of ecological scenarios including mapping communities,^11^ population monitoring^12^ and mapping individuals in small areas.^13^ However, few studies have focused on mapping populations at differing times and places, or the challenges of the homogeneous environment.

An economically important agricultural crop such as winter wheat (*Triticum aestivum L*.) may be significantly impacted by competition from weeds.^14^ Weed species add additional costs to the production of crops by increasing the need for agricultural inputs: e.g. in one national‐scale audit, it was estimated that weeds cost the Australian economy $3.5 billion a year.^15^ Monitoring data can reduce costs by facilitating precision application of inputs such as herbicides, or better‐informed cultural management.^16^ Ecological monitoring depends on being able to locate and enumerate individuals or species within a given environment.^17^ Patches of weeds have shown to be persistent over 10 years, therefore mapping in 1 year represents a potential predictor of future occurrence.^18^ There are many challenges in the mapping of weeds such as their fast growth rates, and highly variable spatial and temporal distributions.^19^ Given the potential value of monitoring data, and the possibility of rapid large‐scale acquisition of data using UAS, there is clear interest by researchers and farmers in applying this technology to measure weed populations.^20^

Despite the potential for data derived from UAS to improve weed management, previous research has highlighted significant issues in their use to monitor weed populations.^6^ Specifically, images and models calibrated to measure weeds in one environment appear to perform poorly when transferred to another. There are several reasons for this limited transferability, for example, variation in weather conditions or different growth stages of the weed or crop. As crop plants grow over the field season their phenology changes, as does that of the weeds.^21^ This results in changes in the spectral properties of the crop and weed species, both in the visible spectrum and beyond.^22^, ^23^ Moreover, common crops are grown in many different varieties, each with their own unique phenology and physiology.^24^, ^25^, ^26^ The statistical methodology of random forests (RF) and a data set of mean pixel values from UAS image plots, as used in our previous study of weed monitoring, does not fully capture the extent of these variations, thus failing to generate highly transferable models.^6^

Supervised machine learning is a statistical method that generates a classification output after being presented with an unclassified input, having previously been trained on data consisting of known inputs and outputs.^27^ All such models are trained using ‘features’. A feature is a numeric representation of the unclassified input. In the case of an image input, these can be engineered by researchers, i.e. texture, colour, shape or they can be abstractly and randomly defined by the model and adapted over iterations. Here, we highlight key network methods that are used in supervised machine learning.

Neural networks conceptually mimic biological neurons in their node‐like structure. Each node is interconnected to others and sends a ‘signal’ if threshold values are passed. Threshold values are tuneable at each node and are adjusted automatically over the course of fitting the model. An important advantage of neural networks is that they can bypass the need for domain knowledge of the data set (feature engineering), allowing more abstract and potentially useful features to be used. This does, however, make the model less interpretable, as the features that are used are selected without logical justification. As with most statistical methods, neural networks perform better when trained on more data.

Convolutional neural networks (CNN) are a type of neural network specifically applied to image data sets. CNN have emerged as the most common, and frequently best performing, model for image classification tasks in the machine learning literature.^28^ CNN learn a sparser connection between regions of an image than traditional neural network models by imposing spatial dependencies upon the pixels in the image.^29^ This may be of use when analysing weed distributions because these are spatially dependant.^30^, ^31^, ^32^ CNN do not use user‐defined features such as colour, shape or texture to learn from the data. Instead CNN create abstract feature maps and then through training/iterations, assign importance to different feature maps^33^ representing different states in the image. These components of a CNN make them well‐suited for mapping weed populations, but the underpinning model correspondingly harder to interpret. Spatial information is retained, and automated abstract feature identification can identify common aspects among the classes of data that human feature selection would otherwise miss.^34^

Here, we investigate how images collected from UAS can be classified using CNN to predict weed densities in unseen images. We explore how data engineering can be undertaken to improve the results and account for the heterogenous nature of the environment. We also investigate the seasonal effects of mapping on our ability to correctly predict weed densities by comparing our models between years and the week of survey, thus addressing key limitations from past literature. Finally, we assess true out of sample predictions of CNN models to assess their transferability across populations.

## 2 MATERIALS AND METHODS

### 2.1 Description of data set

We studied *Alopecurus myosuroides* (black‐grass) in populations of *Triticum aestivum L*. (winter wheat). Some 1.9 million hectares of wheat is cultivated per year in the UK, making it the most widely grown crop, with *A. myosuroides* becoming a significant problem throughout the UK.^35^

Our field sites were part of an ongoing study by the Black Grass Resistance Initiative (BGRI) into herbicide resistance levels in the weed nationally. We surveyed 102 new fields across the arable regions of the UK. Late season monitoring (13 June to 12 August in 2016 and 2017) was chosen because previous work shows that the weeds are distinguishable from the surrounding wheat crops at this time.^6^ This represents a BBCH weed growth stage of 87–89.^36^

Fields were subject to a range of differing management practices, across farms from 80 to 3000 ha. The populations of black‐grass had previously been measured in fields using the methodology developed by Queenborough *et al.* and Hicks *et al.* 3, 35 to estimate plant density states in a plot. Plots of 20 × 20 m were chosen as this allowed large amounts of contiguous ground‐truthed data on the densities of black‐grass in a field to be collected. The average field was 8 ha with 110 plots per field, depending on the varying extents of the field. Five ordinal density states of black‐grass were denoted: absent, low, medium, high and very high, (0, 1–160, 161–450, 451–1450 and 1451+, plants per 20 m^2^ respectively). This method allows for multiple observers to be used, enabling large spatial scales to be covered with minimal misclassification error between observers.

### 2.2 UAS platform

A widely available commercial UAS platform was chosen to allow for low entry costs and high repeatability. We used the 3DR solo UAS (‘Solo ‐ The Smart Drone | Commercial Drone Platform.’ https://3dr.com/solo-drone/. Accessed 11 January. 2018.) because it permits third party imaging systems to be attached and operated. The Parrot Sequoia (‘Sequoia ‐ MicaSense.’ https://www.micasense.com/sequoia/. Accessed 11 January. 2018.) was chosen as the imaging sensor because it has been specifically designed for use with UAS. This sensor records images in four discrete calibrated spectral channels: green 550 nm (*f*_g_), red 660 nm (*f*_r_), red‐edge 735 nm (*f*_re_) and near infrared 790 nm (*f*_n_) at 1.2 Mp. The sensor possesses a ‘sunshine sensor’ that standardized against variable lighting conditions over the course of a flight by continuously recording the light conditions in each spectral channel and then automatically calibrating the outputs to the absolute values.

All flights were carried out following UK rules and regulations controlling the use of UAS for scientific research. Flights were conducted within 2 h either side of solar noon to reduce the effect of sun angle. The optimum flight parameters to cover each field in the minimal amount of time were a flight height of 100 m and an image overlap of 60%.^37^ Each flight generated thousands of subfield scale images that are stitched together to create a single orthomosaic image, encompassing an entire field using relatively few ground control points. For this Agisoft Photoscan was used. This software also creates vegetation indices (VIs) from the individual bands of the sequoia. The average ground sample distance (GSD) of all the flights was 8.27 cm pixel^−1^.

Of the 102 fields that were flown, 76 generated data of high enough quality to analyse. Fields that were not suitable for analysis were discarded for the following reasons: poor image quality, significant image stitching artefacts and sensor failure.

The calibrated spectral channels of the sequoia sensor allow for VIs to be calculated for each pixel. VIs are used because they reduce multiband observations to a single numerical index.^38^ We used the green normalized differential vegetation index (GNDVI; Eqn 1) to classify images:

(1) $$\text{GNDVI}=\frac{\;f_{n}-f_{g}}{f_{n}+f_{g}}$$

All subsequent references to the data, refer to the GNDVI data set (see Table A5 in the supporting information for statistical measurements of the GNDVI dataset).

Our choice to base our analysis on GNDVI is because high biomass crops such as wheat cause saturation of chlorophyll levels in the red wavelength, resulting in poor performance when using the normalized differential vegetation index (NDVI; Eqn 2).^39^

(2) $$ND\mathrm{VI}=\frac{f_{r}-f_{n}}{f_{r}+f_{n}}$$

Previous studies have focused on the NDVI owing to its correlation with plant vigour and growth.^40^ However, when needing to discriminate between invasive populations, vigour and growth rates, NDVI has been shown to be uninformative in cases of high saturation of a spectral channel.^41^ Analysis based on UAS imagery has often overlooked this feature of NDVI, but it is recognized in satellite remote sensing work.^42^, ^43^, ^44^

The ground‐truthed density data were overlaid on each georectified orthomosaic using GIS packages in R. The orthomosaic maps were split into 20 × 20 m subplots, each relating geographically to the ground‐truthed observations. This creates a data set of images at the 20 × 20 m scale, on which our subsequent analysis area is based. The resulting image data set consists of 12 313 unique measurements of black‐grass at a 20 × 20 m scale covering the full range of black‐grass densities. The densities are not evenly distributed, however. The breakdown as follows: Absent = 14.5%, Low = 53.1%, Medium = 17.3%, High = 8.2% and Very High = 6.9%.

### 2.3 Modelling approach and metrics

We used a CNN to train a classifier on our black‐grass image data. The model structure was taken from one of the top performing methods on the industry standard image database, ImageNet,^45^ called GoogLeNet.^34^ Although we use the structure of GoogLeNet, it is important to note that we do not use the pretrained model weights and biases that allowed the model to score so highly on ImageNet. Here, we highlight four common components of our chosen model framework, which are then stacked together with other components such as batch normalization and dropout to create a variety of different network structures:

1. Convolution. The convolutional step involves extracting features from an image while maintaining their spatial context, by using a filter to pass over an image and computing the dot product to create a generalized feature map.
2. Addition of non‐linearity. Non‐linearity is introduced into the feature maps by applying a rectified linear unit (ReLU), this speeds up the training process when compared with tanh/sigmoid activation functions. This means that model convergence will occur with a lower computational cost.^46^
3. Pooling. Pooling of the feature map is used to reduce dimensionality. This reduces the parameter number in the network, a key stage in preventing overfitting. Pooling also makes the network more stable to distortions in the training images.^47^
4. Fully connected final layer. This combines all the neurons of the previous layer and applies an activation function to determine the final classification of an image. The most common form of activation function is SoftMax and the predictions always sum to 1.^48^

CNN have been applied successfully to many data sets similar to ImageNet through a process known as transfer learning, whereby only the weights of the connected final layer of a pretrained model are altered.^49^ We do not use the process of transfer learning because our proposed data set is significantly different from that of ImageNet. Instead, we use the GoogleLeNet structure and independently train all layers of our model.

Three data sets are needed to model a CNN: training, validation and test sets. Each data set comprises pairs of input images and target vectors. Target vectors act as a labelling method and are what the model tries to predict when given a new image. In our example, the input image is a 20 × 20 m image plot and the target vector represents the five different ordinal density states. CNN are trained using a variety of parameters. From our initial exploration of the modelling, we settled on using the following as our standards: a decaying momentum beginning at 0.1 and halving every 32 000 steps as our optimizer, categorical cross entropy as our loss function, and a batch size of 128.

We report, where appropriate, three metrics for our models; these are multiclass AUC, Cohen's kappa and weighted Cohen's kappa. AUC refers to the area under the receiver operating characteristic (ROC) curve, that is the true positive rate (sensitivity) against the true negative rate (specificity). AUC is used for its ability to differentiate between two groups, and is equal to the probability that the classifier will rank a randomly chosen positive example higher than a randomly chosen negative example.^50^ AUC values range from 0 to 1. We plot a diagonal line from (*x* = 0, *y* = 1) to (*x* = 1, *y* = 0) known as the line of equality or the random chance line.^51^ Points that fall below this line represent non‐informative models where random classification would perform better. For the *x*‐axis in our AUC plots we use 1 – Specificity.

The categorical predictions of a model and ground‐truthed observations can be viewed as different raters. This allows us to assess the degree to which they agree or disagree and utilize Cohen's kappa statistic^52^ (Eqn 3):

(3) $$\kappa =\frac{p_{o}-p_{e}}{1-p_{e}}$$

Where ρ_o_ is the observed agreement and ρ_e_ is agreement due to chance. This results in a range from 1 indicating complete agreement between raters, to 0 indicating that agreement is only due to random allocation and −1 indicating complete disagreement.

AUC and kappa do not consider the ordinal structure of our data, with observations ranging from Absent to Very High in incrementing ordered categories. Therefore, an observation of Absent and a prediction of Low is closer to agreeing than if the prediction were Very High. We therefore used weighted Cohen's kappa (Eqn 4):

(4) κw=1−∑ki=1∑kj=1ωijxij∑ki=1∑kj=1ωijmij

Where κ is the number of categories, and ω_ij_, χ_ij_ and *m*_ij_ represent the weight from the matrix. This allows us to count disagreements differently.^53^ The weighted kappa is on the same scale and distribution as the base Cohen's kappa. We use a squared weighting matrix of 1, 4, 9, 16 and 25 ranging from agreement to significant disagreement, to penalize significantly wrong agreements.

### 2.4 Model refinement: data balancing

We checked the performance of the model in several respects. First, we analysed the effect of balancing the data in terms of the distribution of observations among density states. This is important because the data set is heavily weighted towards the Low density state, comprising over 50% of the data set. Such imbalanced distributions can lead to lazy or biased classifiers, whereby the model can default to predicting the majority class but will nevertheless still score well in many metrics such as error or accuracy rate. To investigate this, we created balanced data sets and use metrics as outlined above. In our data set, the Very High class had the smallest representation with only 565 examples in the training set. We therefore randomly sampled 565 of each remaining density states, to create a balanced training set of 2825 images. The same balancing process was repeated for the validation and testing data sets resulting in 800 and 575 images, respectively.

### 2.5 Model refinement: data cleaning

**Figure 1:**
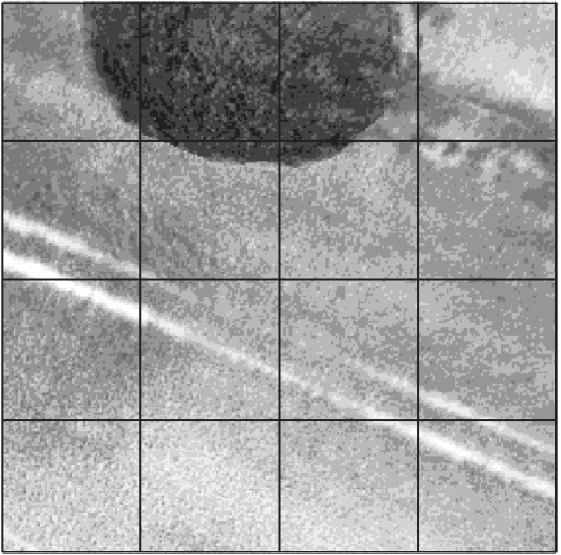
Example of a Very High, 20 × 20 m plot with significant non‐black‐grass ‘artefacts’, reducing the signal in the image coming from the Very High level of black‐grass that was observed on the ground in this plot. The grid overlay represents the subsampling methodology used to break each image into 16 smaller representations of the entire plot. The subplots are referenced by their position relative to the bottom left‐hand corner (1,1) and top right‐hand corner (4,4).

It is important to consider the quality of imaging data. Specifically, many of our 20 × 20 m aerial plots contain ‘artefacts’ that were not accounted for in our ground observations. Figure 1 shows examples of three such types of artefacts. In Fig. 1, an overhanging tree, the tramline and the field hedgerow in the top right‐hand corner introduce significant noise into the image that does not represent either wheat or black‐grass. It is this excess noise/uncategorized data that we aimed to remove.

To achieve this, we subsampled each individual 20 × 20m plot into 16 smaller images. Figure 1 demonstrates the outline of this subsampling grid. This yielded a data set of 197 008 images. We then manually examined this data set and set aside all subsamples that we determined to contain artefacts. In the case of Fig. 1, only two subplots of ‘pure wheat’ remained, (1, 2) and (1, 3), which were subsequently used in what we refer to as the Clean data set. This created a Clean data set of 101 907 images and an Artefact data set of 95 101 images. The training and test sets were the same as the previous experiments, but now ‘cleaned’. We use the Clean and Artefact data sets to build models and predict on the test data of the other data set, e.g. clean model on artefact test data, and *vice versa*. This allows us to test the influence of data cleaning.

To make a comparison with our ground observations, we must upscale the subplot predictions back to the 20 × 20 m scale at which ground observations were recorded. There is often variation in density within each plot, but this is not recorded. In a hypothetical situation this could mean that the model is fitting the subplot test data perfectly, but then being penalized because we are unable to ascertain the observed level of black‐grass in that specific subplot, only the entire 20 × 20 m plot. We therefore take the median prediction from each subplot of one 20 × 20 m plot as the model observation. This gives us a prediction of only the areas of the image with wheat and/or black‐grass in them, at a scale that allows for comparison with our ground‐truthed data.

### 2.6 Model transferability: field level cross validation

To test out‐of‐sample/new field performance, we conducted leave‐one‐field‐out cross validation (LOFO‐CV) trails and created 76 models, i.e. one per field. Each model was trained using the baseline model parameters and cleaned upscaled subplots from all the fields. One field was withheld from the training data set to become the test set in each new model. We report back metrics at field level (i.e. not 20 × 20 m plot level) because not all fields have the full five density states present.

### 2.7 Modelling workflow: baseline model

Having created the relevant data sets for each question, we trained a model using our standard parameters. We began the analysis with a simple baseline test of how the models perform when 10% of the entire data is randomly selected as the test set. The model was then used to predict the ground‐truthed observations of the relevant test set. We then calculated all relevant metrics and plot a ROC curve where appropriate. This assessed the performance of the CNN and established a baseline against which further analysis could be benchmarked. We investigated the effect of data balancing, data engineering and LOFO‐CV against the baseline model.

To account for possible differences owing to variation in the date or survey or between years, we grouped the LOFO‐CV models by years with 38 and 43 fields in 2016 and 2017 respectively, and took the mean values of the AUC for each year. Each field season lasted 6 weeks and averaged the same number of fields each week. Consequently, we grouped the LOFO‐CV models by week and took the mean values of AUC. Owing to the design of our field season, we begin in the south and move north over the course of the season, so latitudinal effects will also be present but are not accounted for.

## 3 RESULTS

### 3.1 Baseline model

**Figure 2:**
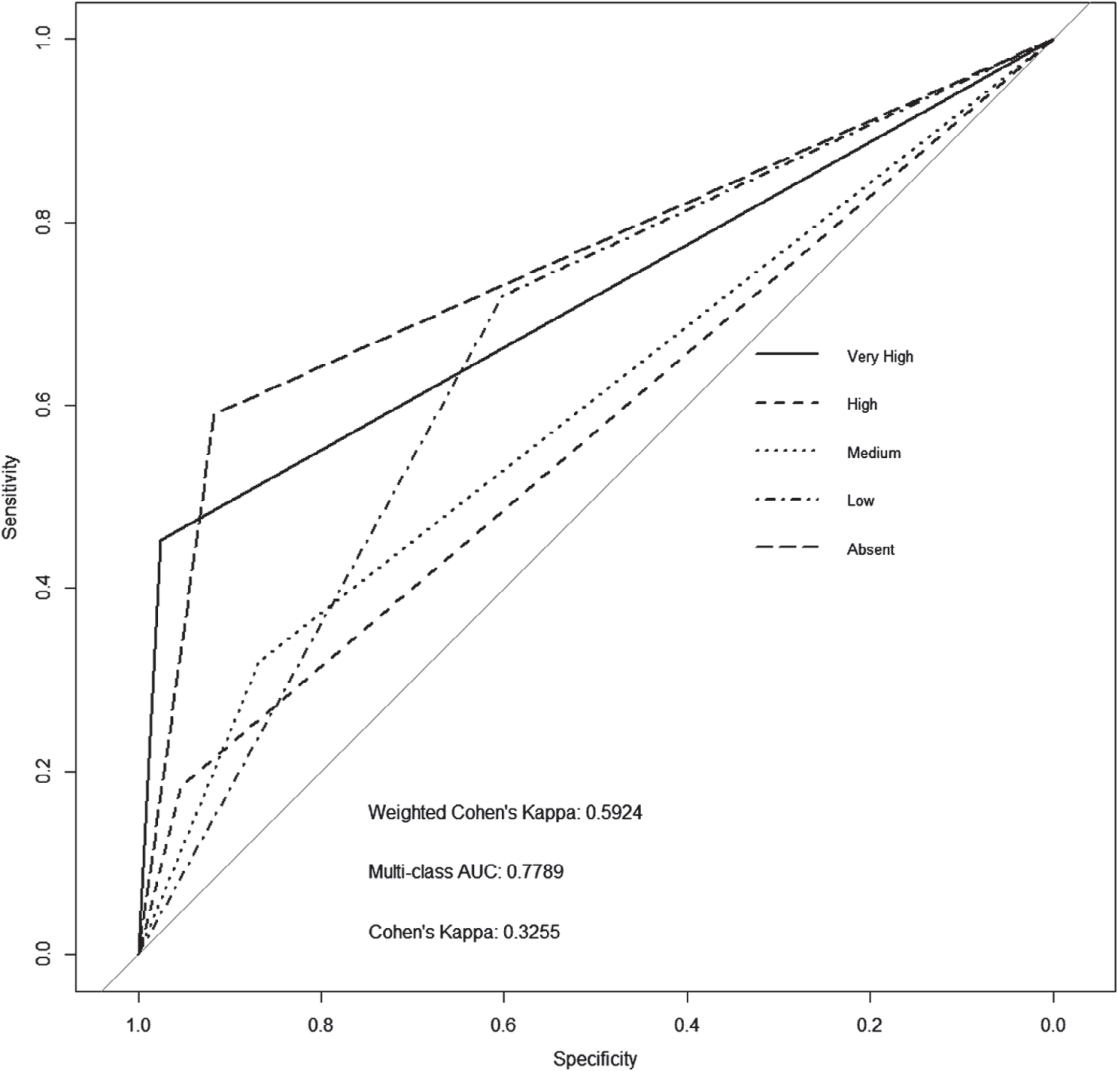
Baseline, receiver operating characteristic (ROC) plot of a convolutional neural network (CNN) trained using 90% of the data set and used to predict the multiclass black‐grass density state of the completely withheld random 10% of data.

We find that the baseline model gives an AUC of 0.78, a weighted kappa of 0.59 and an average misclassification rate across all states of 17.8%, as seen in Fig. 2. We see that the Very High and Absent density states show the AUCs closest to *x* = 1, *y* = 1. This means that these density states are easier to distinguish for the model than the states in between.

### 3.2 Data balancing

**Figure 3:**
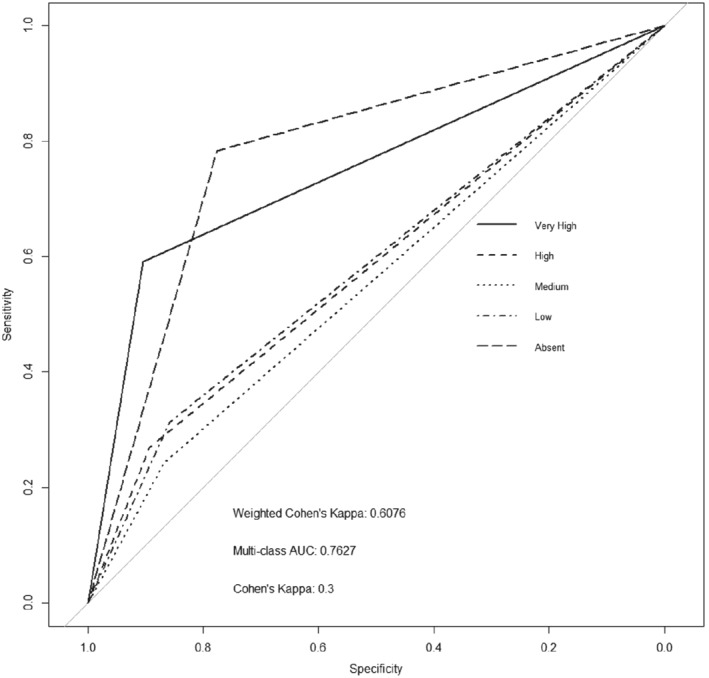
Receiver operating characteristic (ROC) plot of a convolutional neural network (CNN) trained using 90% of the balanced data set used to predict the multiclass black‐grass density state of the completely withheld random 10% of balanced data.

The same training and evaluation parameters were used to train a model for the data in which the proportions of the density states were balanced. We see that by balancing the data set we slightly reduced the AUC and Cohen's kappa of the model (see Fig. 3 for the ROC plot), while increasing slightly the weighted kappa and increasing the misclassification rate to 22.4%. This is most likely a consequence of the reduced number of training samples, leading to a poorer ability of the model to generalize features unique to each class. Tables in the supporting information section A1–A4 present statistical analysis on the differences between curves.^54^ The results in Table A1 (supporting information) show that when the curves from Fig. 2 (baseline model) are compared with those of Fig. 3 (data balanced) all but the Low density state curve are statistically non‐significantly different. Balancing the data set or not does not affect the predictive performance of the models. We therefore continue to use the unbalanced data set for the rest of our analysis.

### 3.3 Data cleaning

**Figure 4:**
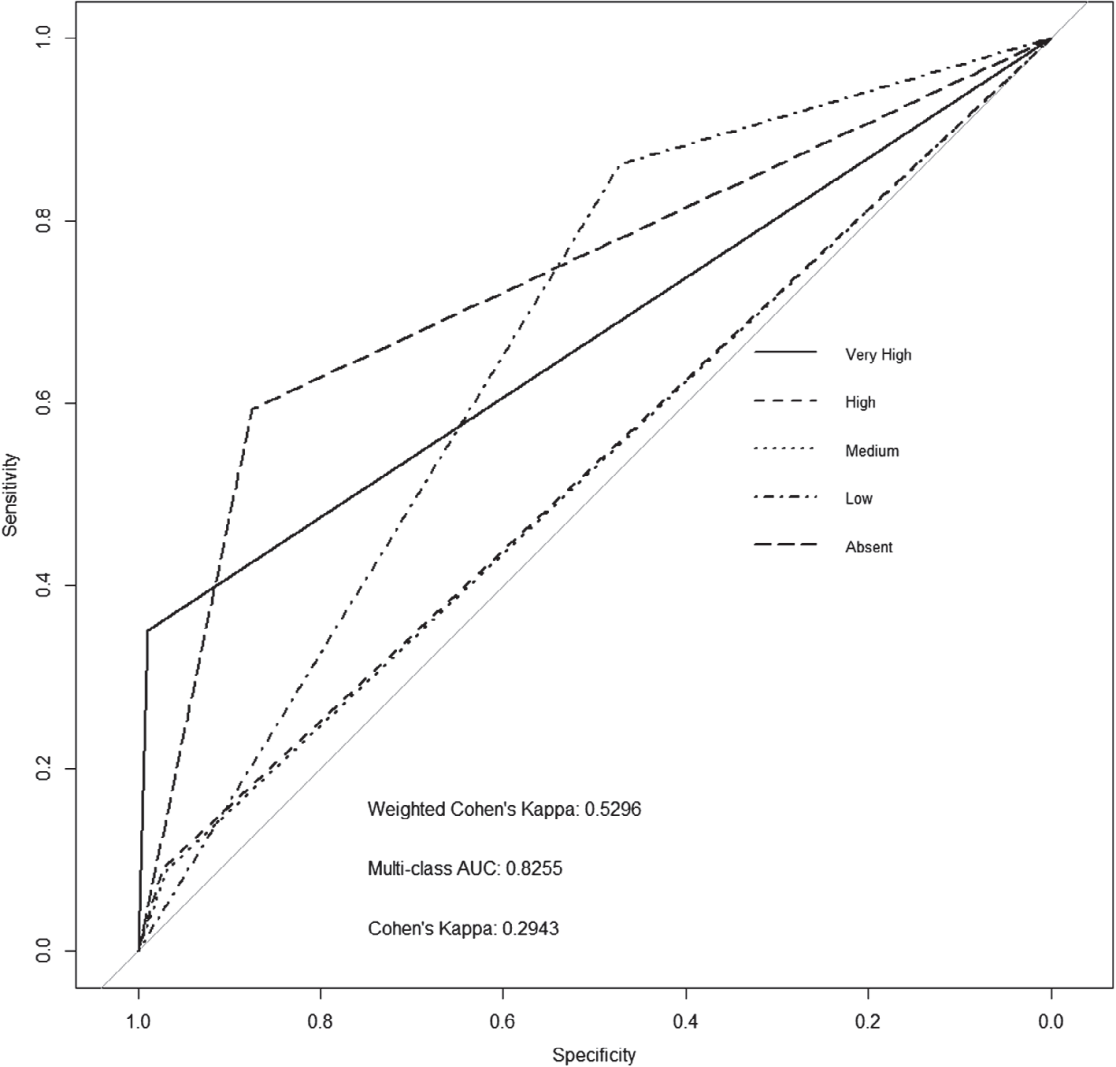
Receiver operating characteristic (ROC) plot of a convolutional neural network (CNN) trained using 90% of the entire Clean subplot data set used to predict the multiclass black‐grass density state of the completely withheld random 10% of Clean data. The subplot predictions are then scaled back up to 20 × 20 m plots for comparison with our ground observations.

To examine how the data cleaning process (Fig. 1) affects our models, a new model was trained using the same parameters as the baseline model, but using the unbalanced, Clean data set. Figure 4 shows that the AUC increased by 4.6%, a significant improvement with a similar misclassification rate to the baseline of 17.5%. Table A2 presents the statistical breakdown of the individual comparisons of AUC to the baseline.

**Figure 5:**
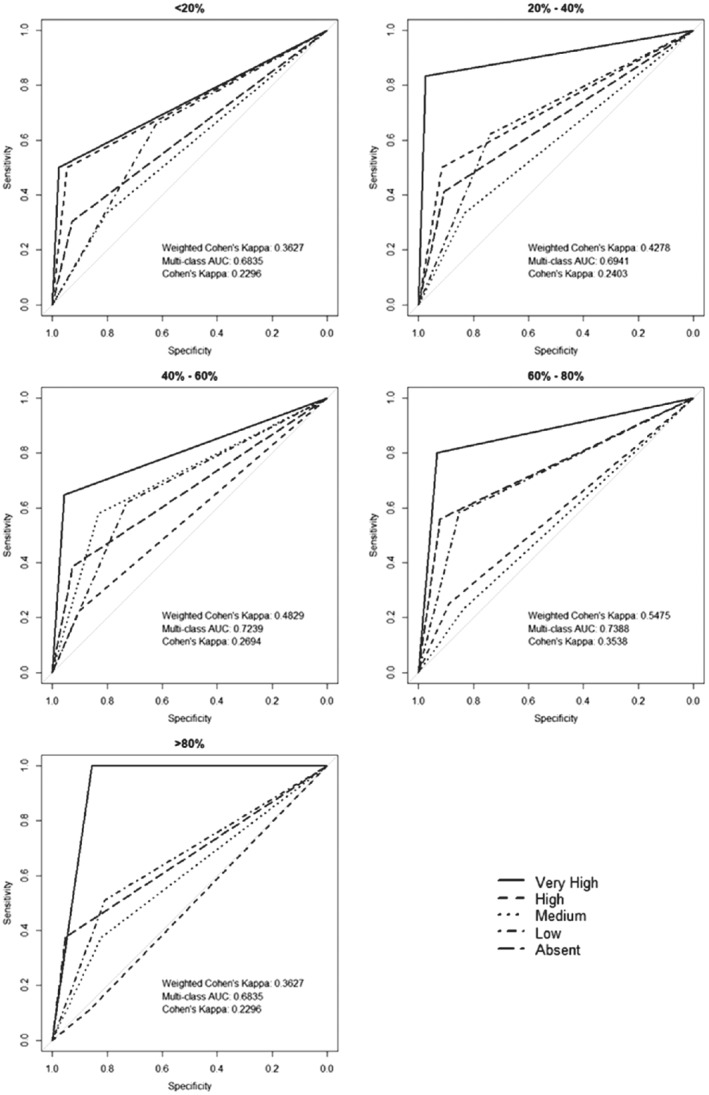
Receiver operating characteristic (ROC) plots showing how the percentage cover of the subplots in the Clean data set affects performance (measured as area under the curve, AUC, and kappa).

The images vary greatly in quality, with some having a large amount of high‐quality coverage, whereas in other cases only a small amount of the image is of good quality. We therefore divided the data set according to only the percentage cover of good quality data of the original 20 × 20 m plots remaining after cleaning, regardless of black‐grass level. Five equal categories of coverage of the 16 subplots, ranging from < 20% (approximately three subplots) to > 80% (13–16 subplots) were established. Looking at the multiclass AUC values for each plot in Fig. 5, we see there is an ∼ 6% difference in the lowest (0.67, < 20%) and highest values (0.73, 60%–80%). We highlight the statistical differences between the categories with the highest and lowest AUCs in Table A3. Showing that although the individual density states lines are not significantly different, the overall graphs are significant in conjunction with Fig. 5.

### 3.4 Analysis artefact data

**Figure 6:**
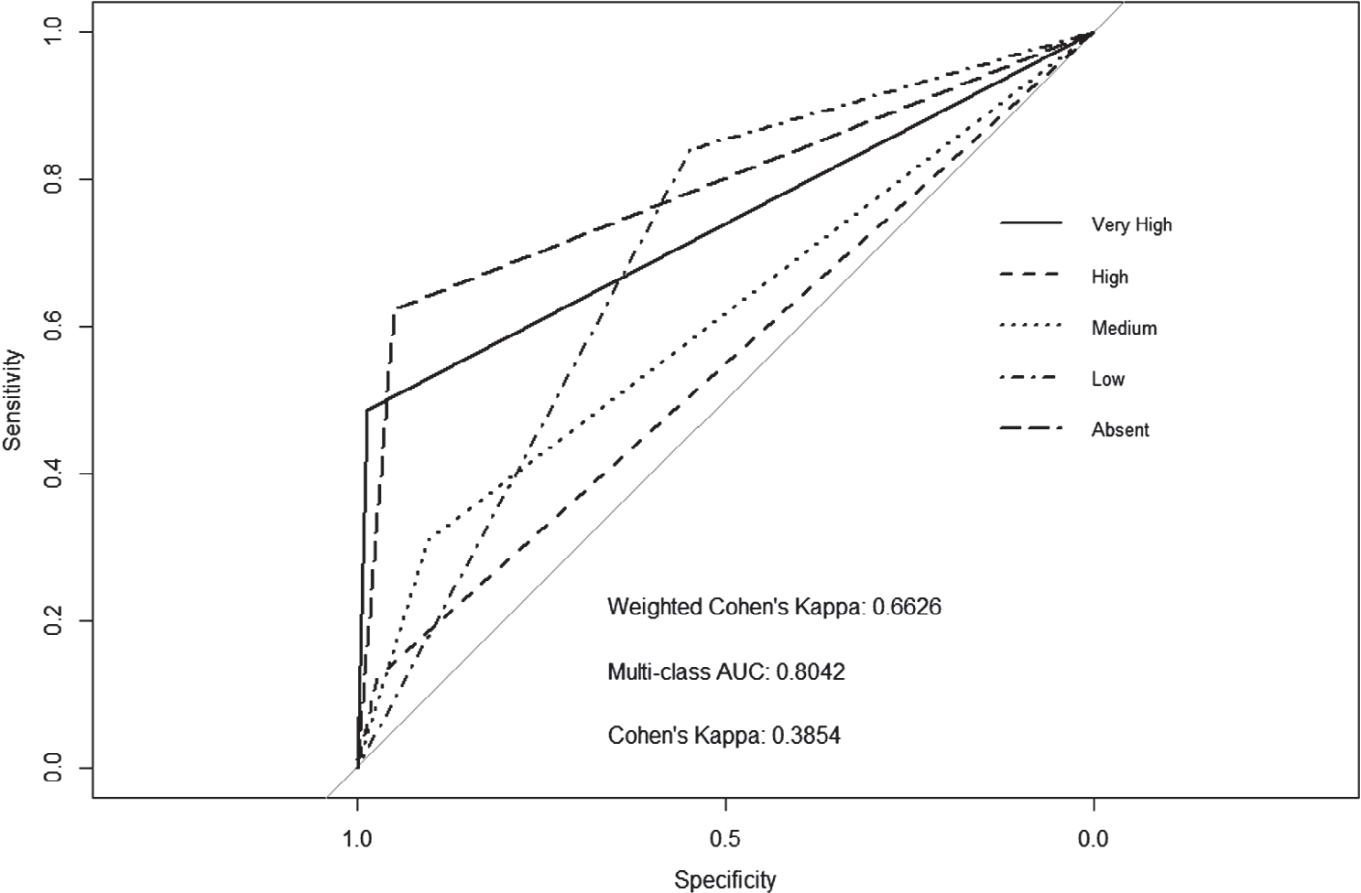
Receiver operating characteristic (ROC) plot of a convolutional neural network (CNN) trained using 90% of the artefact subplot data set used to predict the multiclass black‐grass density state of the completely withheld random 10% of artefact data.

Having shown in Fig. 4 that cleaning and upscaling the data result in improved metrics from the baseline, we next investigated the predictive performance of models fitted to the ‘artefact’ images. To do this, we used the 95 101 artefact images set aside from the training set, predicted on the artefact images from cleaning the test data and then upscaled. Figure 6 suggests that the artefact plots still have features within them that allow us to classify black‐grass as accurately as the Clean model (Fig. 4). It also shows that with a higher weighted kappa and lower misclassification rate of 15.5%, it does better at not making large ordinal disagreements, e.g. Very High observation *versus* Absent prediction, when compared with the Clean model. The Clean model predicted Absent when a Very High was observed in 8.75% of cases, compared with the artefact model predicting only 6.3% of such cases.

**Figure 7:**
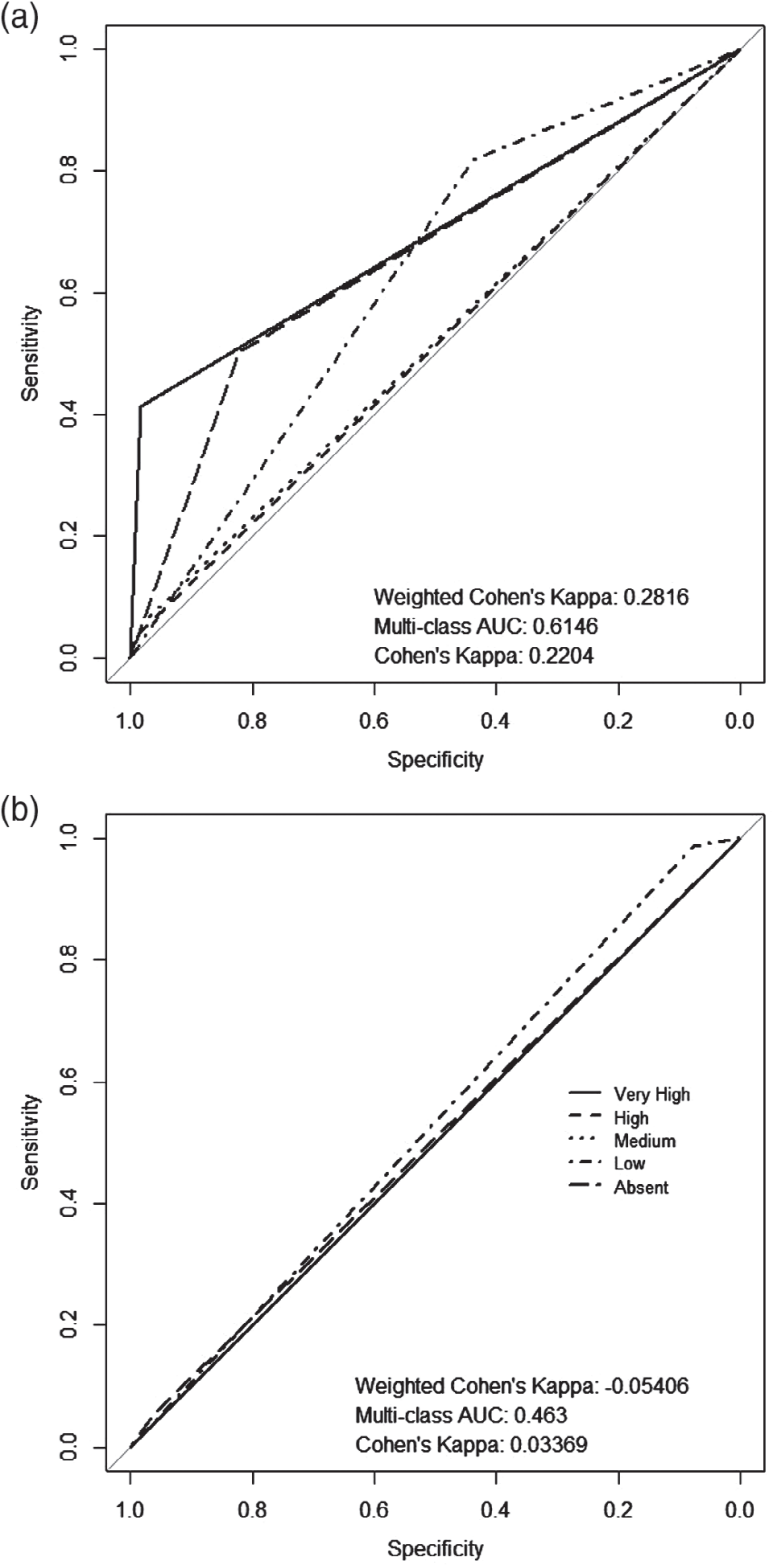
(a) Receiver operating characteristic (ROC) plot of a model trained using the Clean training set, then used to predict the five density level states in the artefact test set. (b) ROC plot of a model trained using the artefact training set, then used to predict the five density level states in the cleaned test set. The predictions are upscaled to plot level.

As shown in Fig. 7, the clean model can predict the black‐grass levels in the Artefact data set with some degree of accuracy, with an AUC of 0.61 and misclassification rate of 17.1%. However, the model for the artefact data is not able to predict the clean test data set accurately, with an AUC of 0.463, a misclassification rate of 42.1% and the AUC for all density states were significantly different as shown in Table A4. This suggests that the features used by the artefact model are not conducive to black‐grass identification. Therefore, the features in the model for Fig. 6 must not be directly related to black‐grass. This also suggests that our manual screening of the data may have been overly strict, and we are thereby missing data that could increase the ability of the model to generalize features for the identification of black‐grass.

### 3.5 Out of sample predictions: LOFO‐CV

Here, we examine the true out of sample prediction for the data set. In all our previous models we used an initial random 10% as our test data set, as described in our initial test set. Therefore, the model has been trained on a large sample of each individual field, allowing it to generalize features specific to that field, making it more sensitive to outliers. Thus, our reported results to date are not truly out of sample and may have limited repeatability in further studies, even when using the standardized data collection methodology described here.

**Figure 8:**
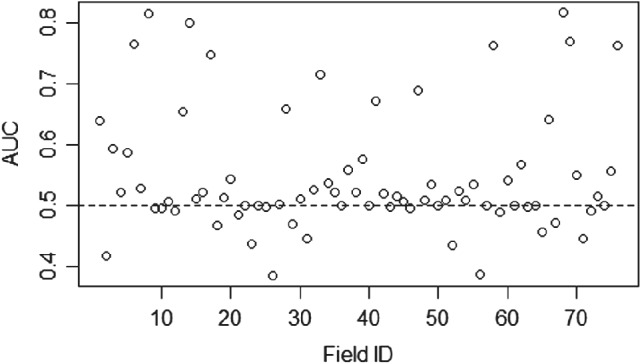
Area under the curve (AUC) of each field's out of sample prediction. Each point represents a separate model that was trained on all but the Field ID in question which is used as the test set. Field ID is a randomized ordering of the field names across both survey years.

Figure 8 shows that the mean AUC of the fields is 0.54 with a range of 0.38–0.81. This means that LOFO‐CV predictions for these models are frequently no better than random. The kappa metrics were not used here because most of our out of sample fields did not contain the full range of black‐grass densities and so are penalized for lack of agreement when there are no observations of a level.

### 3.6 Temporal effects

**Figure 9:**
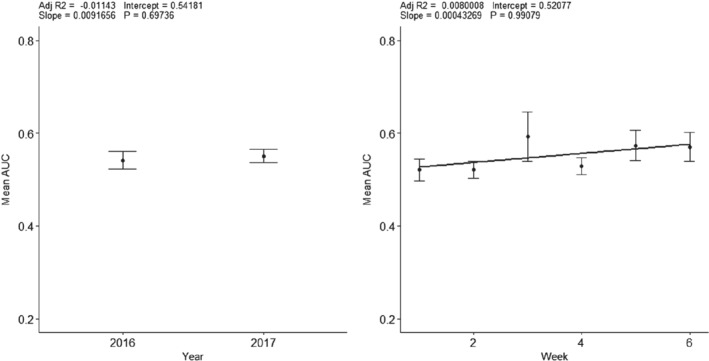
(a) Mean area under the curve (AUC) for every model in each year. (b) Mean AUC for every model in each week.

To investigate temporal effects on the results of our out of sample predictions, we studied whether the year or the week we visited the field had any effect on the AUC. Figure 9 shows the mean and standard errors of the AUC for each year and week. Neither year nor week has a significant effect on the model performance measured by the AUC of the model, with adjusted *R* ^2^ values of −0.011 and 0.008 respectively. This means that the temporal variation in the time surveying has not influenced our results.

## 4 DISCUSSION

We set out to predict distributions of weed densities using UAS imagery and CNN. We have devised a standardized and repeatable UAS data collection methodology, applied it over multiple years across the major arable areas of the UK and utilized data engineering techniques to increase the quality of our data sets. Although the weeds were shown to be detectable, it is by no means a simple task, because both species are grasses with many similar traits. Our main conclusion is that data engineering increases the performance of our metrics the most, relative to other methods attempted when given a sample of known states in a field. Increases in performance such as these are not common for CNN in the computer vision literature. There was no evidence that temporal factors such as year or time of sampling affects the performance of the out of sample predictions.

However, when predicting on fields with no previous ground‐truthing (i.e. true out of sample data), the success as revealed by our metrics was highly variable. This may be due to the problem of data set shift.^55^ Data set or covariate shift occurs when there is a change in the distribution of the classes between the training and test data sets. We know from our ground observations that on an individual field‐by‐field basis, it is rare to find fields with the full five density state distribution and there are no cases where all five are present in an equal distribution. One way of counteracting this issue in the literature is by constructing a density estimation of the labels in the test data set and reweighting the training data set accordingly.^56^ This approach is not applicable in a fully automated UAS system for the prediction of density states, because it is still dependant on ground‐truthed observations from skilled observers.

Our study is the first to use repeated UAS surveys and deep learning statistical methodology to assess the impact of the significant heterogeneity in conditions across time and space on automated monitoring of weed densities. Anderson and Gaston^57^ outline many areas in which UAS can be used in ecology and emphasize the need for temporally resolved studies, allowing for scale‐appropriate measurements using UAS that can be at user‐defined times and locations. This is a change in precedent from remote sensing work using satellite data, where data were available only at set times, resolutions and spectral frequencies. However, many previous studies using UAS have focused on repeated visits to one single site over time^58^ or multiple sites at one time point.^59^ The use of trial plots in some studies does allow for a more detailed assessment of certain variables.^60^ However, in real world applications of methodologies and management decisions developed under these controlled settings, much more spatial and temporal variability when applied in agronomic use cases will be encountered, thus reducing the transferability and scope of the studies.^61^ Therefore, our focus on using only ‘live’ uncontrolled agronomic scenarios does result in reduced reported metrics, but allows our work to be applied with a more realistic understanding of the results that would be seen in the field.

Neural networks have previously been used and compared with other statistical methods, to classify the state of weed populations at a range of spatial scales.^62^, ^63^, ^64^ Barrero *et al.*,^13^ trained a neural network with a user‐defined texture feature derived from NDVI to identify a weed species among a single rice paddy. They reported a 99% precision on test data, with no reported recall score. This is most likely an overstatement of the model performance and approach. However, this study focused on only the binary classification issue of presence/absence of a weed, a much simpler and less informative on‐farm metric, and considered only predictions from a single field at a single time point, suggesting that the performance is being overstated with no LOFO‐CV being attempted. It is to be expected that our metrics (AUC, Cohen's kappa and weighted Cohen's kappa) are lower than the equivalent ones reported in the neural network study, due to our focus on multiple fields spanning a wide variety crop conditions and for the more advanced use of density state predictions. Therefore, our results are more representative and transferable than these studies due to our LOFO‐CV analysis, for methodologies involving UAS and machine learning to map weed populations going forward. However, our results indicate a more extensive and controlled analysis of the transferability of models is still needed.

The process of manually screening the data sets for artefacts is a slow and non‐reproducible or scalable task. In the future, we propose to train a classifier to automatically partition an entire data set into clean and artefact sections. This approach is comparable with work that quantifies the data quality of video using a CNN.^65^ This would allow us to expand our analysis into other VIs by improving and standardizing the data processing pipeline.

With the Artefact data set predicting to the same if not higher standards in our metrics than the Clean data set, it stands to reason that a composite modelling approach could be undertaken to channel the clean and artefact subplots to their respective models and then recombined at the upscaling stage. This is a concept similar to ensemble‐based classifiers, where multiple differing model types are trained on the same data set and aggregate their predictions for the test set.^66^ Our approach described here would use this concept but instead of differing model types on the same data set, we propose the same model on differing data sets and aggregating their predictions. This would reduce the amount of data loss and combine the differing feature sets of the models to aid in the detection of arable weeds.

### 4.1 Concluding remarks

We have demonstrated here how data engineering of UAS imagery and use of CNN can be used to classify weed densities. We highlight the methodological improvements resulting in increased prediction accuracy compared with past research using a variety of metrics, statistics and data collection procedures that provide a more detailed assessment of true model performance. All our models apart from the LOFO‐CV are composed of a random 10% of individual subplots for the test set. This means that the models will have most likely been exposed to some in‐field examples of the test set, and therefore can generate features that are specific and not generalized to detection of the weed. We can conclude that when considering only the images of a new field and no other data, we cannot be highly confident in the ability of most of our models to map the black‐grass in the field. Although we do not show a significant improvement in LOFO‐CV testing with no apparent factors that make an individual field be predicted well or poorly. We believe that the robustness of this evaluation procedure is a greater estimation of real‐world predictive value when compared with past studies, which consequently overestimate their applicability. Therefore, the methodology set out in this paper represents a new standard in the area of weed mapping with UAS due to the expanded capabilities of data collection, statistical methods and evaluation procedures.

## Supporting information


**Table S1.** Non‐Equal dataset AUC's compared to the Equal datasets. Used (54) to test the statistical difference of the AUC of each Density state.
**Table S2.** Non‐Equal dataset AUC compared to the Cleaned dataset.
**Table S3.** Worst performing bracket AUC from data quality testing (20% <)(AUC 1) compared to the best performing AUC bracket (60% – 80%) (AUC 2).
**Table S4.** Clean model AUC's from predicting on the artefact dataset compared to the Artefact model AUC's from predicting on the clean dataset.
**Table S5.** Statistical measurements of the GNDVI pixel values for each vegetation group.
