# Supplementary material for: Testing the ability of unmanned aerial systems and machine learning to map weeds at subfield scales: a test with the weed Alopecurus myosuroides (Huds)
Source: Pest Manag Sci. 2019 May 21;75(8):2283–94. doi: 10.1002/ps.5444 (PMC6767585; doi:10.1002/ps.5444)
Supplement: Supplementary file 1 — Table S1. Non‐Equal dataset AUC's compared to the Equal datasets. Used (54) to test the statistical difference of the AUC of each Density state. Table S2. Non‐Equal dataset AUC compared to the Cleaned dataset. Table S3. Worst performing bracket AUC from data quality testing (20% <)(AUC 1) compared to the best performing AUC bracket (60% – 80%) (AUC 2). Table S4. Clean model AUC's from predicting on the artefact dataset compared to the Artefact model AUC's from predicting on the clean dataset. Table S5. Statistical measurements of the GNDVI pixel values for each vegetation group. [file PS-75-2283-s001.docx]

**Appendix**

Table 1 Non-Equal dataset AUC’s compared to the Equal datasets. Used (54) to test the statistical difference of the AUC of each Density state.

| Density State | AUC 1 | AUC 2 | D | p-value |
| --- | --- | --- | --- | --- |
| Abs | 0.75 | 0.77 | -0.94 | 0.34354 |
| Low | 0.66 | 0.58 | 2.87 | 0.004032 |
| Med | 0.59 | 0.55 | 1.48 | 0.138393 |
| High | 0.56 | 0.58 | -0.46 | 0.643204 |
| V High | 0.71 | 0.74 | -1.00 | 0.313908 |

Table 2 Non-Equal dataset AUC compared to the Cleaned dataset.

| Density State | AUC 1 | AUC 2 | D | p-value |
| --- | --- | --- | --- | --- |
| Abs | 0.75 | 0.73 | 0.76 | 0.445291 |
| Low | 0.66 | 0.66 | -0.34 | 0.727911 |
| Med | 0.59 | 0.52 | 3.72 | 0.000193 |
| High | 0.56 | 0.53 | 1.81 | 0.069353 |
| V High | 0.71 | 0.67 | 1.34 | 0.177991 |

Table 3 Worst performing bracket AUC from data quality testing (20% <)(AUC 1) compared to the best performing AUC bracket (60% - 80%) (AUC 2).

| Density State | AUC 1 | AUC 2 | D | p-value |
| --- | --- | --- | --- | --- |
| Abs | 0.61 | 0.74 | -2.25 | 0.023968 |
| Low | 0.64 | 0.71 | -1.47 | 0.140146 |
| Med | 0.56 | 0.53 | 0.23 | 0.813781 |
| High | 0.72 | 0.56 | 1.12 | 0.261717 |
| V High | 0.73 | 0.86 | -0.70 | 0.479862 |

Table 4 Clean model AUC’s from predicting on the artefact dataset compared to the Artefact model AUC’s from predicting on the clean dataset.

| Density State | AUC 1 | AUC 2 | D | p-value |
| --- | --- | --- | --- | --- |
| Abs | 0.66 | 0.51 | 12.22 | 2.43E-34 |
| Low | 0.62 | 0.53 | 11.62 | 2.94E-31 |
| Med | 0.52 | 0.5 | 4.70 | 2.56E-06 |
| High | 0.51 | 0.5 | 2.88 | 0.003877 |
| V High | 0.69 | 0.5 | 9.26 | 1.92E-20 |

Table 5 Statistical measurements of the GNDVI pixel values for each vegetation group.

| GNDVI | Mean | Standard Deviation |
| --- | --- | --- |
| Black-grass | 0.336 | 0.007 |
| Winter wheat | 0.304 | 0.011 |
